# A Silicon-Based ROTE Sensor for High-Q and Label-Free Carcinoembryonic Antigen Detection

**DOI:** 10.3390/mi15050580

**Published:** 2024-04-27

**Authors:** Luxiao Sang, Haojie Liang, Biao Zhao, Runze Shi, Aoqun Jian, Shengbo Sang

**Affiliations:** 1 Shanxi Key Laboratory of Micro-Nano Sensors & Artificial Intelligence Perception, College of Electronic Information and Optical Engineering, Taiyuan University of Technology, Taiyuan 030024, China; 2 Key Laboratory of Advanced Transducers and Intelligent Control System, Ministry of Education, Taiyuan University of Technology, Taiyuan 030024, China; 3 Shanxi Research Institute of 6D Artificial Intelligence Biomedical Science, Taiyuan 030031, China

**Keywords:** resonant optical tunneling effect, optical biosensor, carcinoembryonic antigen detection, quality factor

## Abstract

This paper presents a biosensor based on the resonant optical tunneling effect (ROTE) for detecting a carcinoembryonic antigen (CEA). In this design, sensing is accomplished through the interaction of the evanescent wave with the CEA immobilized on the sensor’s surface. When CEA binds to the anti-CEA, it alters the effective refractive index (RI) on the sensor’s surface, leading to shifts in wavelength. This shift can be identified through the cascade coupling of the FP cavity and ROTE cavity in the same mode. Experimental results further show that the shift in resonance wavelength increases with the concentration of CEA. The biosensor responded linearly to CEA concentrations ranging from 1 to 5 ng/mL with a limit of detection (LOD) of 0.5 ng/mL and a total Q factor of 9500. This research introduces a new avenue for identifying biomolecules and cancer biomarkers, which are crucial for early cancer detection.

## 1. Introduction

Tumor markers are of significant practical importance in cancer screening, diagnosis, and therapeutic efficacy evaluation [1,2,3]. Among the numerous tumor markers, CEA has been the most thoroughly studied since its discovery in 1965 [4]. In healthy adults, CEA levels in plasma are very low [5] but are aberrantly expressed in many human cancers, such as colorectal cancer [6], breast cancer [7], gastric cancer [8], pancreatic cancer [9], and lung cancer [10], making the detection of CEA concentrations crucial. Most of the current methods for CEA detection are based on immunoassay techniques, including the enzyme-linked immunosorbent assay (ELISA) [11], electrochemical immunoassay [12], and fluorescence immunoassay [13]. While these immunoassays offer good selectivity, they require complex and expensive instrumentation and skilled operators. Improper handling may also pose risks of radiation damage to humans. Therefore, developing a rapid, efficient, and cost-effective method for CEA detection is especially important for human disease diagnostics.

Optical resonators can confine resonant light in minimal space. Furthermore, the light–matter interaction is greatly enhanced by the multiple round trips of light in a microscale volume, which can be used for high-sensitivity sensing. The most extensively researched microcavities over the past 20 years are whispering gallery mode (WGM) microresonators, which have the advantages of an ultra-high quality factor, a small mode volume, and great sensitivity [14]. WGM optical platforms have already been used for some of the most demanding biosensing tasks, such as detecting single molecules [15,16,17], detecting single virus particles [18,19], and monitoring the structural dynamics of individual proteins [20]. However, due to its diminutive size, it takes a considerable amount of time for the WGM resonator to capture the target analytes, thereby limiting the broad application of WGM microcavities.

Compared with the WGM resonator, the resonant optical tunneling effect (ROTE) resonator is a rectangular resonant cavity with a particular multilayer dielectric structure [21]. The ROTE is derived from the optical phenomenon of frustrated total internal reflection (FTIR), which was first discovered and named by Pochi Yeh [22]. It has the advantages of easy fabrication, high stability, and low cost. In 1999, Hayashi et al. [23] experimentally verified the existence of the resonant optical tunneling effect phenomenon for the first time. The ROTE has been utilized in various optical devices, including optical switches [24] and accelerometers [25]. In 2019, the ROTE was first applied to detect the concentration of various cells [26]. The experimentally obtained cell concentration resolution was about 2.53 nm/(amol/mL) with a detection limit of 1.2 × 10^5^ cells/mL. This sensor device is comparable to the best results obtained by other optical cell sensors but still suffers from a low Q factor.

In this study, the functionality of the ROTE sensor is significantly bolstered by enhancing its Q factor, while new potential applications are explored. The ROTE sensor takes advantage of the low loss of silicon in the infrared band to increase the Q value by a factor of 15. The biosensor was manufactured using self-assembly technology and the covalent coupling method. A promising strategy for CEA detection is proposed based on the variation of the effective refractive index (RI) on the surface of the ROTE biosensor. Key performance parameters such as linear range, specificity, and stability, were critically evaluated.

## 2. Experimental Procedure

### 2.1. Materials

The CEA, Anti-CEA, and Alpha-fetoprotein (AFP) were all purchased from Shanghai Linc-Bio Science Co., Ltd., Shanghai, China, and the Fluorescein isothiocyanate (FITC)-labelled anti-CEA was acquired from Sino Biological (Beijing, China). The cysteamine (CYS), 1-ethyl-3-carbodiimide (EDC), N-hydroxy-succinimide (NHS), phosphate-buffered saline (PBS) buffer (0.01 mol/L, pH = 7.4), and bovine serum albumin (BSA, 99%) were obtained from Solarbio (Beijing, China). Human serum albumin (HSA) was obtained from Shanghai Yugong Biotechnology Co., Ltd., Shanghai, China. All other reagents were analytically pure and were provided by Sinopharm Chemical Reagent (Shanghai, China). The experimental water was ultrapure water (URT-11-10T).

### 2.2. Apparatus

Incident light was provided by a tunable infrared fiber laser (TIFL, TLB-6700, New Focus, San Jose, CA, USA). The light emitted from the laser was regulated by a fiber polarization controller (FPC, KG-PFPC, Conquer Photonics Co., Ltd., Beijing, China). The reflected light was collected by a photodetector (1191-FC-AC, New Focus, San Jose, CA, USA). Finally, the offset of the reflected spectrum was recorded by a digital storage oscilloscope (DSO, GDS-2302A, GWINSTEK, Suzhou, China). Scanning electron microscopy (SEM) images and elemental analysis (EDS) were obtained from a Gemini SEM 300 (ZEISS, DEU). Atomic force microscope (AFM) images were obtained using the NX10 atomic force microscope (Park Systems, Suwon, Republic of Korea). Fluorescence images were obtained with a Vert.A1 (ZEISS, Oberkochen, Germany).

### 2.3. Preparation of Biosensors

The double-sided polished silicon wafers were cut into square slices with a size of 2 cm × 2 cm to serve as the ROTE biosensor platforms. Then, they were ultrasonically cleaned with acetone, deionized water, ethanol, isopropanol, and deionized water for 15 min each, then dried in a nitrogen stream. A layer of gold with a thickness of 50 nm was sputtered on the surface of the cleaned silicon wafers. On the non-sputtering side, a polymer layer (MY-131-series) was spin-coated as the ROTE structural tunneling layer. Finally, they were left at room temperature for 4 h and laminated to the triangular prism using an ultraviolet ray adhesive. These gold-plated sensor platforms were immobilized with anti-CEA to form the ROTE biosensor.

**Figure 1:**
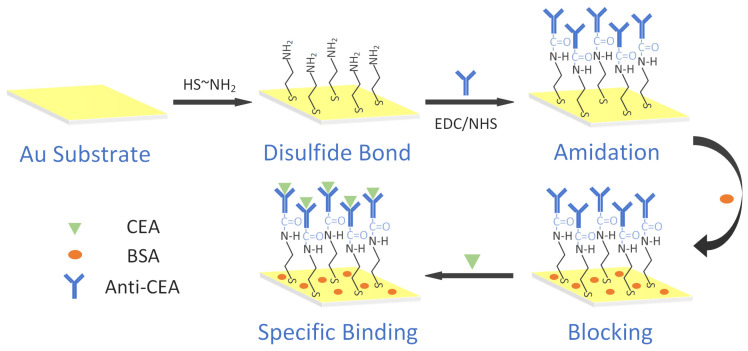
Schematic diagram of the procedure to functionalize the ROTE biosensor.

The functionalization process of the sensor surface is shown in Figure 1. The previously processed sensor platforms were rinsed with ethanol and deionized water and dried under a stream of nitrogen. Then, the sensor platforms were immersed into 40 mmol/L cysteamine, protected from light at room temperature for 12 h, washed with ethanol and deionized water, respectively, and dried under a nitrogen flow. The modified sensors were then activated for 30 min at 37 °C via submerging in a solution of 40 mmol/L EDC and 10 mmol/L NHS (ready-to-use). After washing with deionized water and drying with nitrogen, the activated sensors were placed in 50 μg/mL of the anti-CEA dilution solution, incubated for 60 min at 37 °C, and washed with the PBS buffer and deionized water, respectively. The anti-CEA-loaded sensors were submerged in the BSA solution and incubated for 30 min at 37 °C to block the non-specific binding site. They were then rinsed three times with the PBS buffer and deionized water and dried in a nitrogen stream. Finally, the prepared ROTE biosensors were stored in a refrigerator at 4 °C until use.

### 2.4. Optical Signal Detection

**Figure 2:**
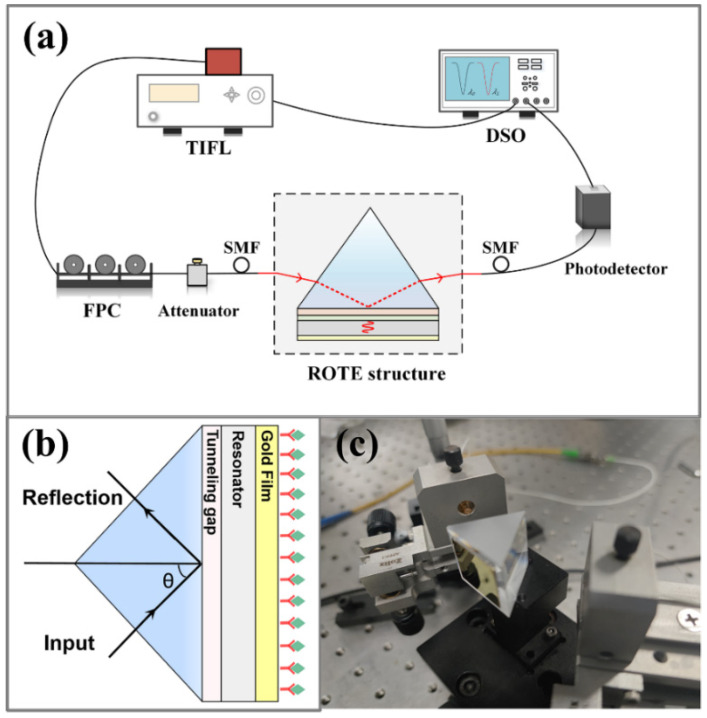
(**a**) Schematic representation of the ROTE biosensor measurement system. (**b**) The ROTE structure close-up. (**c**) A photograph of the ROTE structure.

The experimental setup is shown in Figure 2a, and the tunable infrared fiber laser (TIFL) was directly connected to the fiber polarization controller (FPC) to ensure that the outgoing light was S-polarized [27]. After the incident light signal passed through the FPC and attenuator, it entered the collimated fiber and incident at a set angle of incidence θ. The reflected light can be collected and detected by a photodetector. The ROTE sensing structure was fixed on the electronically controlled rotary stage to precisely adjust the angle of incidence and then cooperate with the optical displacement platform to realize the collimation of the incident light (as shown in Figure 2c). The collected reflected light was converted into electrical signals by a photodetector and connected to a digital storage oscilloscope (DSO) to obtain real-time, accurately reflected spectra.

The optical resonator sensor mainly uses the change of resonant wavelength for sensing detection, and the resonant wavelength is primarily determined by the cavity length of the resonator and the effective RI of the mode.

When the antibody on the surface of the ROTE sensor binds to the antigen, it causes a change in the effective RI of the resonant cavity. To maintain the resonant state, the growth of effective optical length of the resonator results in a shift in its resonant wavelength. Therefore, the RI change can be obtained by detecting the shift of the resonance peak. And the concentration of CEA can be correlated to the change in resonant wavelength observed in the reflection spectrum.

## 3. Results and Discussion

### 3.1. Characterization of the Surface Functionalization of the ROTE Biosensor

**Figure 3:**
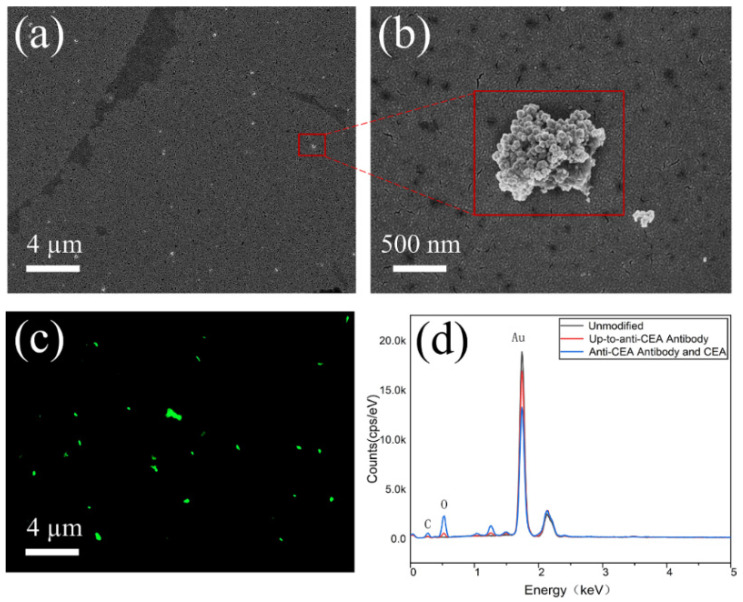
(**a**) SEM image of the biosensor surface after anti-CEA immobilization. (**b**) Details of the anti-CEA. (**c**) The fluorescence image of the sensor coated with anti-CEA. (**d**) The EDS spectrum of the unmodified immunosensor surface (black), after anti-CEA immobilization (red), and after the binding of anti-CEA and CEA (blue).

SEM and EDS characterized the sensor’s surface in terms of morphology and elemental composition. Following the anti-CEA modification on the sensor surface, as depicted in Figure 3a, a plethora of spherical aggregates become visible on the sensor surface. A detailed close-up is presented in Figure 3b to provide a vivid representation of the anti-CEA details. Figure 3d compares the EDS energy spectra of the sensor surface before and after the functionalized modification. Given that anti-CEA is fundamentally an envelope glycoprotein abundant in carbon and oxygen, the concentration of these elements increases markedly, whereas the amount of gold diminishes, when both CEA and anti-CEA are implemented as modifications on the surface.

Fluorescein isothiocyanate (FITC) can react with amino groups to produce yellow-green fluorescence [28]. Therefore, the presence of the protein can be demonstrated by staining the protein with FITC. The surface of the sensor after modification was observed using fluorescence microscopy with 495 nm excitation, as shown in Figure 3c. Many yellow-green fluorescence spots distributed on the sensor surface can be clearly observed, which demonstrates that the anti-CEA was successfully modified on the sensor surface and covers the whole area uniformly. The functional modification of the anti-CEA was achieved by using a combination of self-assembly and a covalent bonding method. These analyses suggest that anti-CEA was successfully immobilized on the surface of the biosensor.

### 3.2. Detection of CEA

For optical sensors, a high Q factor results in sharper resonant peaks and higher sensor resolution. As per the findings of Gansch et al. [29], the Q factor of an optical resonant cavity is influenced by a multitude of factors and the influence can be dissected as follows:

This is example 1 of an equation

(1) $$\frac{1}{Q_{total}}=\frac{1}{Q_{str}}+\frac{1}{Q_{abs}}+\frac{1}{Q_{rad}}$$

where *Q_str_* is determined by the resonant cavity structure, *Q_abs_* is derived from the loss of material absorption, and *Q_rad_* is influenced by factors such as the coupling efficiency and thermal radiation. According to a previous study by our group [26], the ROTE structure has a high *Q_str_*, indicating that the ROTE structure is an excellent resonator. Consequently, minimizing the absorption of the resonant cavity material presents the most straightforward and simplest approach to augment the Q factor. Given that silicon ranks among the materials with the lowest absorption coefficient in the near-infrared band, a meticulously polished silicon wafer was elected to serve as the resonant cavity in our application.

**Figure 4:**
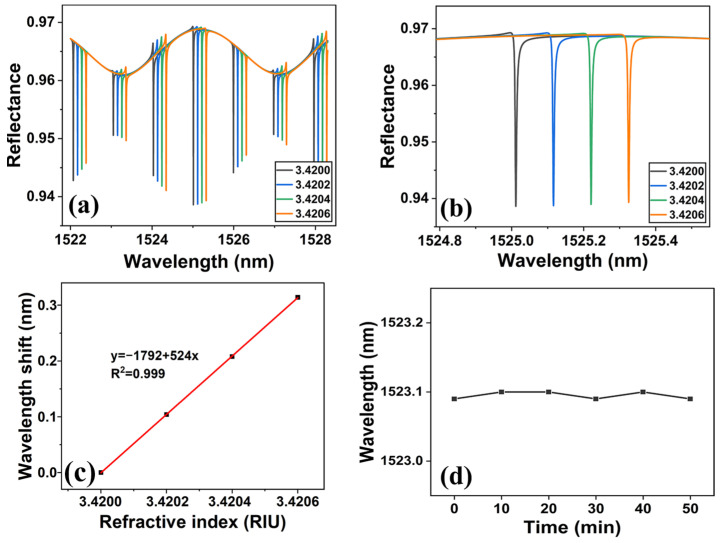
(**a**) The simulated reflection spectrum of the ROTE biosensor. (**b**) A close-up of the resonance wavelength shift (near 1525 nm). (**c**) The calibration curve: the shift of the resonance wavelength with the refractive index change. (**d**) The effect of temperature on resonance wavelength shift during the test.

The transmission matrix method was used to simulate the designed sensing structure, and the results are shown in Figure 4a. It is found that the reflection spectrum of the system is composed of period-distributed ROTE peaks with a flat sinusoidal envelope. The sinusoidal spectral lines appear because of the Fabry–Perot cavity-like structure between the polished silicon wafer and the prism. Consequently, the ROTE-sensing structure proposed in this work can be conceptualized as a cascade coupling of the FP cavity and the ROTE cavity. The existence of the sinusoidal envelope contributes to the distinction between the ROTE peaks of varying modes due to their differential depths. Within such an envelope, the most profound ROTE peak becomes the most distinguishable, and designating it as the marker for the sensing peak facilitates a lucid observation of the wavelength shift. By using the coupling effect, the envelope induced by the FP cavity can help locate the ROTE resonance peak of the same mode. Zooming in on a certain band of the simulated reflection spectrum (about 1525 nm) reveals that the resonance wavelength is red-shifted when the RI of the resonant cavity grows (as in Figure 4b). The relationship between the resonant wavelength shift and RI change is shown in Figure 4c. Thus, the concentration of CEAs can be measured by observing the shift of the resonant peak. It is to be noted that long-term laser irradiation will heat the resonant cavity and may affect its RI. Therefore, at a specific laser power, the same sample was scanned continuously to offset the thermal effect for the measurement. The change of resonant wavelength was recorded every 10 min. As shown in Figure 4d, the resonant wavelength fluctuation in the test is about 0.01 nm. The temperature stability of the ROTE biosensor is excellent.

**Figure 5:**
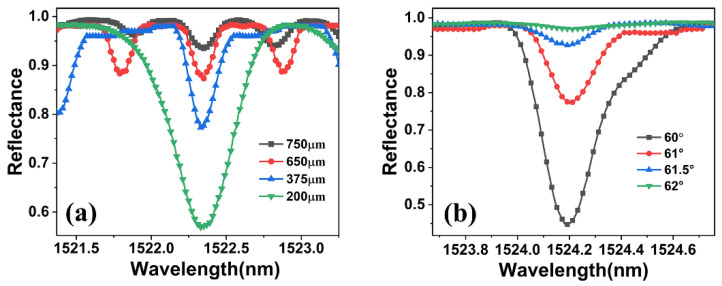
(**a**) Reflection spectra of ROTE sensors with different cavity lengths. (**b**) Reflection spectra of ROTE sensors with different incidence angles.

Moreover, two structural parameters of the ROTE, the cavity length and incident angle, also affect the Q factor. As shown in Figure 5a, the depth of the resonance peak and the free spectral range (FSR) increases significantly with the decrease in the resonant cavity length (for comparison, the ROTE peaks in Figure 5 have been aligned to the same position), but the Q factor tends to become smaller. Figure 5b shows that both the resonance depth and Q factor drop when the incidence angle rises. After balancing the FSR, resonance depth, and Q factor, the cavity length was selected at 375 µm with a 61° incidence angle for wavelength scanning.

**Figure 6:**
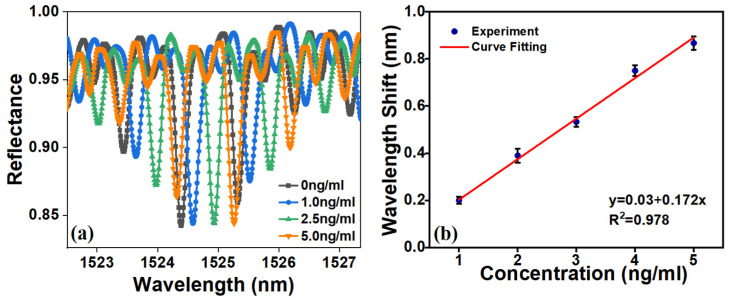
(**a**) The wavelength shifts at different CEA concentrations (1~5 ng/mL). (**b**) The calibration curve: the shift of the resonance wavelength with the change of CEA concentration.

The prepared biosensor was combined with different concentrations of CEA. After incubation at 37 °C for 30 min, the sensor was placed into the experimental test system, and the wavelength shift of the biosensor was monitored and recorded. The response of the ROTE biosensor to the CEA concentration is shown in Figure 6a, from which it can be seen that the wavelength is red-shifted with an increasing CEA concentration.

The calibration curve of the resonance wavelength with the CEA concentrations is shown in Figure 6b. At each concentration, the biosensor calibration experiments were repeated more than three times under the same conditions. Furthermore, *t*-tests incorporating 95% confidence intervals were conducted for the varying concentrations. The resulting *p* value of less than 0.05 provided statistical evidence of a significant change in wavelength. The ROTE biosensor demonstrated a linear response for anti-CEA concentrations within a range of 1 ng/mL to 5 ng/mL. The linearity equation is $y=0.174C_{CEA}+0.03$ (R^2^ = 0.976). The LOD was calculated as 0.5 ng/mL according to Equation (2) [30].

(2) $$L=\frac{3\sigma }{S}$$

where σ is the standard deviation of the blank sensor, and S is the slope of the analytical curve.

As presented in Table 1, various detection methods for the CEA are summarized. the optical biosensor presented in this study is primarily composed of a silicon wafer and a gold film. Key characteristics include a sensitivity of 523 nm/RIU, excellent specificity, a minimum sample volume with a detection limit of 0.5 ng/mL, and an analysis time of 55 s. The detection range is relatively small, but its detection limit is comparable to other methods. In addition, the proposed ROTE biosensor has the advantages of easy fabrication, label-free detection, and a low cost.

### 3.3. Repeatability

**Figure 7:**
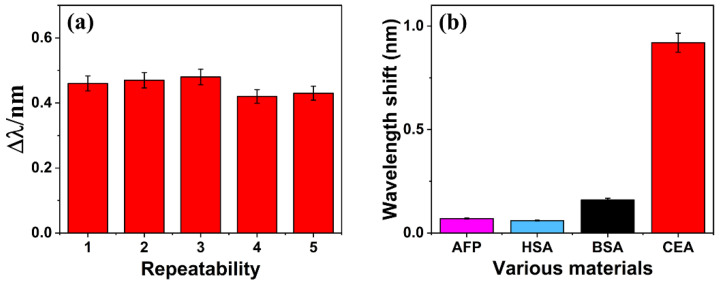
(**a**) A repeatability evaluation of ROTE-structured sensors for detecting 2.5 ng/mL. (**b**) A comparison of resonance wavelength shifts in CEA and three other related proteins based on the ROTE biosensor.

Five sensing chips were prepared under the same experimental conditions and incubated with 2.5 ng/mL of CEA to investigate the reproducibility. As shown in Figure 7a, the wavelength shifts obtained for each sensor were almost the same, with a relative standard deviation of 5.7%, indicating an acceptable reproducibility of CEA detection.

### 3.4. Specificity

The ROTE biosensor was employed for detecting identical concentrations of BSA, AFP, and HSA, as a means to validate the specificity of the tailored sensor. Figure 7b exhibits the response generated by the ROTE biosensor. In detecting CEA, the biosensor resulted in a more pronounced response than that of other biomolecules, indicating the high specificity of the ROTE biosensor for CEA detection. Moreover, the nonspecific adsorption occurring between alternative biomolecules and the sensor engendered a minor shift in wavelength.

## 4. Conclusions

The CEA, as one of the most extensively studied tumor markers, plays a crucial role in the detection of human diseases. However, current immunoassay-based techniques are costly and require specialized operators, highlighting the urgent need for a convenient and quick method for CEA detection.

In this work, we propose a new ROTE-based method for CEA concentration detection. The ROTE biosensor utilizes self-assembly technology, antigen-antibody specific recognition, and the RI-sensitive property of the ROTE structure to achieve the biofunctionalization of the sensor and the detection of CEA. Employing the cascade coupling between the FP cavity and the ROTE cavity aids in pinpointing the deviation of the ROTE resonance peak in the same mode. The sensor response shows that the CEA concentration exhibits linearity in the spectrum of 1–5 ng/mL, accompanied by a corresponding shift in wavelength, with the LOD at a mere 0.5 ng/mL, which is below the lower limit of the normal reference range.

This method could significantly enhance disease diagnostics, tumor screening, and scientific care in modern medicine. Future research should focus on improving the detection efficiency, stability, and reproducibility of the biosensor. One approach involves attaching two-dimensional materials with superior optical properties, better biocompatibility, and a larger surface-to-volume ratio to the sensor surface. Integrating existing machine learning algorithms to train detection results could lead to more efficient and accurate biosensing technologies.

## Figures and Tables

**Table 1:** Comparison of various biosensors for CEA detection.

Method	Detection Range (ng/mL)	Detection Limit (ng/mL)	Reference
Surface acoustic waves (SAWs)	0.2~5.0	0.2	[31]
Electrochemical Sandwich Immunoassay (ESI)	0.5~200	0.5	[32]
Fluorescence	0.4~100	0.316	[33]
Colorimetric aptasensing	4~25	0.19	[34]
Sandwich aptasensor	5~40	3.4	[35]
Colorimetric Immunoassay	1~320	0.37	[36]
ROTE biosensor	1–5	0.5	This work

## Data Availability

Data are contained within the article.
